# HCV Interplay with Lipoproteins: Inside or Outside the Cells?

**DOI:** 10.3390/v12040434

**Published:** 2020-04-12

**Authors:** François-Loïc Cosset, Chloé Mialon, Bertrand Boson, Christelle Granier, Solène Denolly

**Affiliations:** CIRI—Centre International de Recherche en Infectiologie, Univ Lyon, Université Claude Bernard Lyon 1, Inserm, U1111, CNRS, UMR5308, ENS Lyon, F-69007 Lyon, France; chloe.mialon@ens-lyon.fr (C.M.); bertrand.boson@ens-lyon.fr (B.B.); christelle.granier@ens-lyon.fr (C.G.); solene.denolly@ens-lyon.fr (S.D.)

**Keywords:** hepatitis C virus, lipidation, lipoproteins, cell entry, immune escape

## Abstract

Hepatitis C virus (HCV) infection is a major public health issue leading to chronic liver diseases. HCV particles are unique owing to their particular lipid composition, namely the incorporation of neutral lipids and apolipoproteins. The mechanism of association between HCV virion components and these lipoproteins factors remains poorly understood as well as its impact in subsequent steps of the viral life cycle, such as entry into cells. It was proposed that the lipoprotein biogenesis pathway is involved in HCV morphogenesis; yet, recent evidence indicated that HCV particles can mature and evolve biochemically in the extracellular medium after egress. In addition, several viral, cellular and blood components have been shown to influence and regulate this specific association. Finally, this specific structure and composition of HCV particles was found to influence entry into cells as well as their stability and sensitivity to neutralizing antibodies. Due to its specific particle composition, studying the association of HCV particles with lipoproteins remains an important goal towards the rational design of a protective vaccine.

## 1. Introduction

Hepatitis C virus (HCV) was first identified in 1989 and belongs to the *Hepacivirus* genus of the *Flaviviridae* family. HCV infection represents a major cause of chronic liver diseases worldwide, leading to liver fibrosis, cirrhosis and hepatocellular carcinoma. HCV is a small enveloped virus with a single positive stranded RNA (RNA(+)) that encodes a polyprotein that is further cleaved in ten mature proteins: three structural proteins (core, E1 and E2) and seven non-structural (NS) proteins (p7, NS2, NS3, NS4A, NS4B, NS5A and NS5B). Briefly, core corresponds to the capsid protein while E1 and E2 are the two surface envelope glycoproteins. P7 is a small hydrophobic protein involved in assembly and release of viral particles. NS2 is both an auto-protease and a cofactor of assembly. NS3 is both a protease and helicase, and acts with its cofactor NS4A. NS4B and NS5A are proteins involved in the replication of viral RNAs, including the formation of specific membranous rearrangements, and assembly. Finally NS5B is the RNA-dependent RNA polymerase [1].

Since 2014–2017, new treatments based on direct-acting antivirals (DAAs) have emerged. These molecules target three different proteins: the protease activity of NS3, the actions of NS5A in replication and assembly, and the polymerase activity of NS5B [2]. DAAs can now cure most patients; however, there remain major challenges in basic, translational and clinical research [3]. Indeed, HCV is unique among other viruses in that its interplay with lipid metabolism is required for all steps of its life cycle, still making highly instructive the basic research and knowledge gained with this virus. In addition, HCV elimination remains challenging due to possibilities of reinfection, under-diagnosis and poor access to treatments in some countries; hence, making the development of a protective vaccine a priority to achieve eradication of this virus [4]. Furthermore, modelling studies suggest that timely HCV elimination would be facilitated by the combined actions of DAAs and a yet to be developed preventative vaccine [5,6]. Reinforcing the necessity of a protective vaccine [7], the risk of developing hepatocellular carcinoma after treatment with DAAs is increased, suggesting that DAAs can eliminate the virus but not all the consequences of the infection. However, the structure of the HCV virion remains unsolved but is likely unusual, due to its particular lipid composition. While studies with cell culture-grown HCV particles, called HCVcc, have tremendously advanced the knowledge of HCV and host-virus interactions, culminating with new DAAs regimens that cure most patients, many aspects of HCV biology remain ill-defined because of the lack of models fully mimicking the conformation of authentic HCV particles. Thus, a better knowledge of HCV virion morphogenesis remains important for the development of a vaccine as well as for basic virology knowledge. Here, we have discussed recent advances on the mechanism of maturation and lipidation of the HCV particles.

## 2. HCV Particles: A Unique Composition

**Figure 1:**
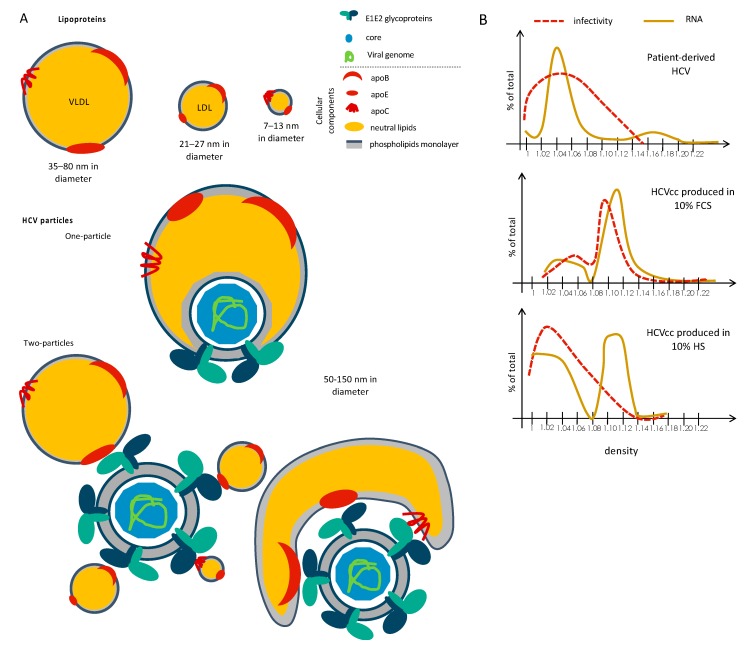
Unique composition and structure of Hepatitis C virus (HCV) particles. (**A**) Schematic representation of lipoproteins and two proposed models of HCV particles, depicted as “one-particle” or “two-particles” models. Viral particles are composed of both cellular (lipids and proteins) components and viral components (proteins and nucleic acids). (**B**) Schematic representation of buoyant density profiles of the infectivity and viral RNA of HCV particles produced in vivo from HCV-infected patients vs. ex vivo assays in the presence of 10% fetal calf serum (FCS) or human serum (HS).

HCV particles were called lipo-viro-particles (LVP) [8] due to the low-buoyant density of infectious particles found between <1.03–1.10, which is particularly low and heterogenous as compared to other enveloped viruses that generally have higher specific infectivity in the high densities. Indeed, in vivo experiments revealed that the low-density HCV particles retrieved from chimpanzees were the most infectious after inoculation in naïve chimpanzees [9,10]. This original property is due to the unique composition of the HCV virion. Besides a classical structure of enveloped virus featuring the E1 and E2 glycoproteins inserted on a lipid bilayer membrane that surrounds the nucleocapsid made by core and RNA(+), HCV particles also contain neutral lipids such as triglycerides (TG) and cholesterol esters (CE) that are presumably located between the two phospholipids layers of its membrane as well as exchangeable apolipoproteins such as apoCIII, apoCI, apoE, and the non-exchangeable apoB apolipoprotein [8,11,12,13,14,15,16] (Figure 1A). Of note, apoE seems better exposed than the viral glycoproteins themselves on the outer of the viral envelope, with a higher copy number of apoE than E2 per particles [12,17], though the stoichiometry of the different virus components within infectious particles remains unknown. The lipid composition of HCV particles is closely related to that of low-density lipoproteins (LDL), very-low-density lipoproteins (VLDL) and high-density lipoproteins (HDL) that are circulating in the blood and responsible for the transport of lipids across the organism (Figure 1A “Lipoproteins”).

Two models of incorporation of lipoproteins within infectious particles have been proposed. The first one is the formation of a true lipo-viro-particle (the “one-particle” model; Figure 1A “One-Particle”), in a way featuring the incorporation of viral particles inside a lipoprotein. The second one is a peripherical association between viral particle and lipoprotein(s) (the “two-particles” model; Figure 1A “Two-Particles”), through non-covalent association of lipoproteins with viral particles that probably occur through interaction between E1E2 and apolipoproteins, especially apoE (see below).

Importantly, although this unique feature of HCV particles has been known for more than twenty years, the processes by which HCV particles associate with neutral lipids and apolipoproteins remain poorly characterized. This is due to (i) the lack of in vitro infectivity of patient-derived particles, preventing the investigation of these particles as well as functional assays with these particles, (ii) the lack of appropriate cellular models allowing both efficient production of infectious particles and secretion of a complete and normal set of VLDL [18,19]. Indeed, Huh-7.5 cells, that are derived from a hepatocellular carcinoma, still remain the best cell type for efficient replication and production of HCV in vitro [20,21], albeit it has lost the capacity of secretion of VLDL [22]. As a result, infectious particles obtained in cell culture (HCVcc) have a higher density and a lower specific infectivity in low density fractions as compared to those derived from patients [12,20,23,24] (Figure 1B). Hence, the HCVcc assay does not allow to fully understand the mechanism of association between HCV particles and lipoproteins components. Due to the lipid metabolism defects of Huh-7 cells, several studies used alternative assays to produce HCV particles; yet, none of them were able to restore the secretion of particles with the same profile than patient-derived particles (see below).

Experimentally, HCV association with neutral lipids is often studied by measuring the buoyant densities of viral particles from equilibrium density gradients made by ultracentrifugation, which reveals that this interaction induces virion shift to lower densities. However, changes in their density profile may also reflect other alterations of the composition of particles; for example, the amounts of glycoproteins or apolipoproteins can modulate the density of particles. Furthermore, it is important to keep in mind that most experiments reported to date, including proteomic or lipidomic analysis of virions, have been performed in a context that does not allow a full lipidation of particles, such as for HCV particles derived from patients. Because of this, the analysis of HCV density gradients at equilibrium needs to be examined carefully.

## 3. HCV Assembly and Connection with Lipoprotein Synthesis Pathway: Association to Lipids within the ER Lumen?

### 3.1. Lipoprotein Biogenesis Pathway

**Figure 2:**
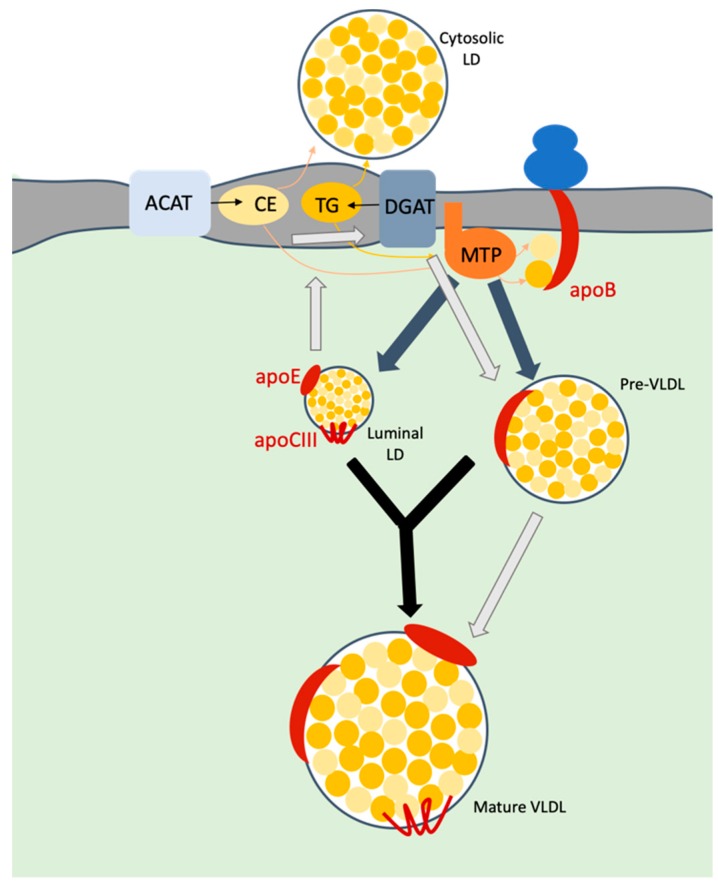
Model of VLDL biosynthesis. Upon translocation of apoB into ER lumen, microsomal transfer protein (MTP) transfers triglycerides (TG) and cholesterol esters (CE) to this apolipoprotein. TG mainly originate through the action of the diacylglycerol acyltransferase-1 (DGAT1) protein whereas CE are derived from the action of acyl-CoA cholesterol acyltransferase (ACAT). Both neutral lipids accumulate within the membrane bilayer and are recruited to form either cytosolic or luminal lipid droplets (LDs). MTP is responsible for the formation of a pre-VLDL as well as of luminal LDs (grey arrows). The maturation of VLDLs is thought to occur either via the fusion of the pre-VLDLs with luminal LDs (black arrows) or through a cascade of hydrolysis/re-esterification of TG from LDs (light grey arrows). Mature VLDL are then secreted in the extracellular medium.

Due to the particular composition of HCV particles, several reports addressed the involvement of VLDL biogenesis pathway (reviewed in [25]) in assembly of HCV virion. The formation of mature VLDLs occurs within hepatic cells (Figure 2). Briefly, VLDLs are synthetized after translocation of apoB100 in the ER lumen and its subsequent interaction with the microsomal transfer protein (MTP). This protein is responsible for the transfer of neutral lipids, i.e., TG and CE as well as phospholipids to apoB, during and after its translocation to the lumen, which induces the release of pre-VLDL particles. On the other hand, it seems that MTP is also responsible for the formation of luminal lipid droplets (LDs) that incorporate apoE and apoCIII at their surface. Acyl-CoA cholesterol acyltransferase-1 and -2 (ACAT-1 and -2), which are located at the ER membrane, are believed to be the main factors allowing the generation of the pool of CE [26] used for VLDL biogenesis, whereas diacylglycerol acyltransferase-1 and -2 (DGAT-1 and -2) catalyze the last step of TG synthesis at the ER membrane [27,28]. Next, VLDLs become mature through incorporation of additional triglycerides on pre-VLDLs and their association with exchangeable apolipoproteins such as apoE, apoCIII. The exact mechanism for this second step remains unclear although a proposed mechanism relies on the fusion of pre-VLDLs with luminal LDs harboring apoE and apoCIII at their surface. An alternative hypothesis was also proposed, consisting of the hydrolysis of TG of luminal LDs that is followed by re-esterification as TG and then addition to nascent lipoproteins [29]. To complement this latter mechanism, it was also shown that cell-death inducing DFFA-like effector B (CIDEB) [30,31] and/or proteins on cytosolic LDs were involved in the maturation of VLDLs, but again, the mechanisms involved remain to be elucidated [29].

### 3.2. Key Role of apoE in HCV Assembly and Maturation

Concerning HCV morphogenesis and its interplay with the VLDL biogenesis pathway, it was first proposed that E1E2 secretion is dependent on the assembly of apoB-containing lipoproteins [32]. Next, using different approaches, apoE was found to be required for HCV assembly [16,33,34,35,36,37,38]. Indeed, knock-down or knock-out of apoE decreased the production of infectious particles. Dissection of the different steps of assembly revealed that apoE acts after assembly, probably at a post-envelopment step. Complementing this observation, it was shown that ectopic expression of human apoE induces the production of infectious HCV particles in non-hepatic cell lines, such as 293T kidney cells [35,39] and murine hepatic cells [40]. In addition, apoE was shown to interact with E2 [16,34], through a robust association that is resistant to stringent lysis conditions [34]. Finally, apoE was shown to interact with NS5A, suggesting an association of apoE with HCV proteins during the first steps of assembly [34,38,41]. Yet, a direct interaction between apoE and NS5A is unlikely to occur since apoE is located in the luminal side of the ER membrane whereas NS5A is exposed to the cytosol. Of note, CIDEB was proposed to mediate this interaction [42]; yet, like for NS5A, CIDEB is directed towards the cytosolic side of the membrane. One possibility to reconcile these observations is that there may be no direct protein-protein interaction (i.e., between apoE and NS5A or CIDEB), but rather, a tight co-localization of these proteins in a specific membranous sub-compartment, as inferred by the fact that apoE-NS5A interaction is relieved upon more stringent lysis [34].

In agreement with the importance of apoE in HCV morphogenesis, it was also shown that core and apoE co-traffic within the same vesicles of the cell secretory pathway, again suggesting that association with apoE and particles occurs during the assembly and/or envelopment steps [43,44].

Regarding the involvement of apoE in the acquisition of neutral lipids by the HCV virion, there are controversial results concerning the buoyant density distribution of infectious particles produced in apoE down-regulated cells as they appear slightly shifted toward higher densities [34] or remain unchanged [33,38]. Since the density profile of physical particles, as assessed by RNA or core quantification, remained unaffected in these reports [34,36,38], this suggested a modulation by apoE of the specific infectivity rather than of virion lipidation.

### 3.3. Involvement of Other Actors in HCV Assembly/Maturation

Several studies have highlighted the role of cellular factors in assembly and maturation of HCV particles.

First, it was shown that the long-term culture of HCVcc-infected Huh-7.5 cells in media containing human serum led to restoration of secretion of complete VLDLs and the production of infectious particles of low density that are associated to apoB, thus resembling those derived from patients [18], indicating that the VLDL assembly machinery is important for the lipidation of particles.

Besides apoE, apoB and MTP were also shown to be important for HCV assembly [41,45], although their exact implication remains controversial [33,46]. Indeed, some studies indicated that inhibition of MTP, and, as a consequence, of apoB secretion, affects the assembly of infectious particles only when apoE secretion is inhibited, hence suggesting that MTP and apoB might not be essential for HCV assembly [46]. However, the role of MTP in luminal LDs formation and the presence of apoE at their surface could be essential for HCV assembly.

As mentioned before, CIDEB was also found to be involved in HCV assembly by allowing the interaction of apoE with NS5A and, subsequently, the recruitment of apoE at the assembly site and its association with HCV particles, perhaps to promote their assembly. In addition, the down-regulation of CIDEB was found to result in particles with a slightly higher density [42].

Annexin A3 (ANXA3), a phospholipid binding protein, was also identified through a quantitative lipid droplet proteome analysis of HCV-infected cells as a factor involved in HCV maturation linked with the viral usage of apoE [47]. Moreover, in HCV-infected cells lacking ANXA3, the secreted infectious particles are detected at higher densities. Since apoE secretion was specifically decreased in ANXA3-KD infected cells, this suggested that ANXA3 could play a role in HCV maturation by modulating apoE secretion.

The chemical inhibition of ACAT proteins, that blocks production of cholesterol ester, resulted in a slight shift of infectious particles to higher densities, which again reflected the connection between maturation of infectious particles and the different factors involved in VLDL biogenesis [48,49].

### 3.4. Proposed Model for Association with Lipoprotein during HCV Assembly

**Figure 3:**
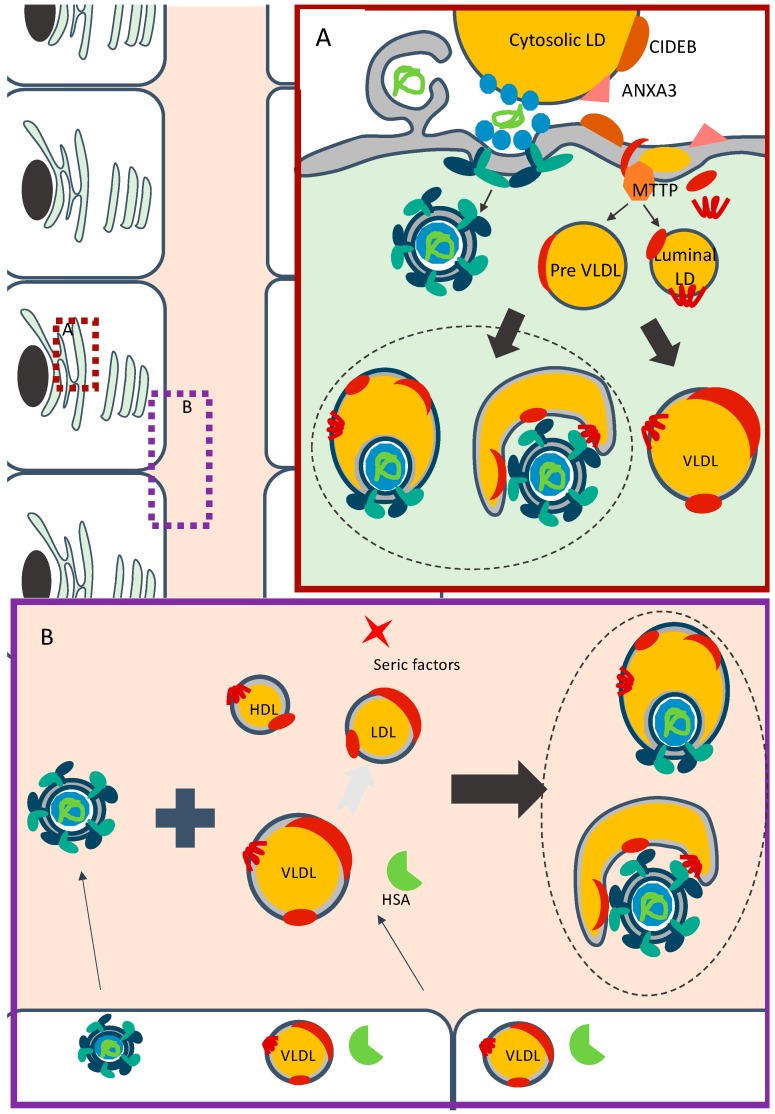
Models for association of HCV with lipoproteins components. (**A**) Model for association of HCV particles within infected cells. HCV replication takes place in double-membrane vesicles (for simplicity, the non-structural proteins are not depicted). The early stages of assembly occur in close proximity between replication organelles and cytosolic LDs. Following or during their budding, HCV particles may associate with preVLDLs or with mature lipoproteins, and are secreted through a specific route. (**B**) Model for HCV particles maturation in the extracellular milieu. HCV particles, already associated or not with lipoproteins components, are secreted by infected hepatocytes. In the extracellular milieu, these particles could interact with lipoproteins and serum factors, which are secreted or not by the hepatocytes. With the help of human serum albumin (HSA) or alternative unidentified serum factors, HCV particles associate with the three classes of lipoproteins to generate mature infectious particles.

Based on all these reports, a possible mechanism of HCV assembly and maturation by lipidation infers that, after budding into the ER lumen, amounts of CE and TG are incorporated into HCV particles in the same manner than for lipoproteins via the action of MTP and/or via fusion of viral particles with pre-VLDLs or with luminal LDs (Figure 3A). Importantly, while most of these observations were derived from studies on the role of apoE, which can exist in both lipid-associated and lipid-free forms, they do not directly address how viral particles incorporate neutral lipids within infected cells. In addition, since most experiments were performed in conditions for which HCV production, from Huh-7.5 cells, does not allow full lipidation of particles (Figure 1B), it is difficult to assess the involvement of the aforementioned identified factors in the maturation of infectious particles and their association with lipids. Finally, it is not always clear if and how cellular proteins are involved in the early steps of assembly vs. in the maturation in particles.

## 4. HCV Particles and Lipoproteins Association: A Dynamic Process in the Extracellular Medium

Over the last few years, several reports indicated that although HCV virion lipidation as well as its association with apolipoproteins could occur in the producer cells, the density of extracellular viral particles and thus, their association with lipids and apolipoproteins can be modulated and evolve independently of the infected cells. Indeed, several evidences suggested that lipidation does not necessarily occur intracellularly. Specifically, when HepG2 hepatoma cells, thought to represent more mature hepatocytes than Huh-7 cells, are induced to produce normal, apoB-containing VLDLs, they allowed the secretion of infectious particles that are biophysically and biochemically similar to those produced from Huh-7.5 cells [50]. Likewise, HCVcc particles produced in PHH, which produce normal VLDLs levels, have higher specific infectivity owing to the viral RNA peak that coincides with the fractions of highest infectivity at densities of 1.10–1.11, which nonetheless remains well above those of patient-derived HCV [51]. In the same manner, HCV particles grown in PHH-xenograft mouse models that release normal human apoB and apoE levels display coincident infectivity and viral RNA peaks at 1.06–1.11 [19], which, again, are higher than for patient-derived HCV. All these results suggest that there is no direct correlation between the secretion of mature lipoproteins and lipidation of particles.

### 4.1. The Redundant Role of Apolipoproteins

It was recently shown that apolipoproteins harboring amphipathic α-helices such as apoCI, apoAI, apoAII, apoCII and apoCIII could replace apoE for HCV assembly in apoE KO Huh-7.5 cells [33,52], suggesting the redundant role of apolipoproteins in HCV assembly. Likewise, alternative secreted proteins structured with amphipathic α-helices like human cathelicidin [53] as well as the Erns and NS1 viral factors from the Flaviviridae members [54] can also replace apoE for assembly of HCV particles. This suggested that apoE per se may not be critical inside the producer cells, but rather, that its structure in amphipathic α-helix and its preferential interaction with viral proteins explains its major and preferential role in HCV assembly.

While apoE, or alternative factors carrying amphipathic α-helices, seem necessary at the assembly stage of viral particles, perhaps by inducing the membrane curvatures required for virion budding [55], apoE also contributes to other viral functions, such as lipidation, immune escape and cell entry, owing to its specific interaction with virions. Yet, there are evidences that this interaction may occur while the viral particles are formed and even secreted. Indeed, some reports indicated a differential regulation of the secretion of apoE vs. HCV particles since factors inhibiting apoE secretion did not affect release of HCV particles [56,57]. This indicates that apoE may not follow the same secretion route as infectious particles, which argues for an association at a later stage of the viral life cycle, like for example in the extracellular medium after virion egress.

### 4.2. Evidences for Extracellular Maturation of HCV Particles

HCV particles associated with apoB48, the apoB form expressed at the surface of chylomicrons, have been detected in sera from infected patients [58]. Since HCV is not produced by enterocytes, in contrast to chylomicrons, this result suggested a post-egress maturation of viral particles. Further evidence for an extracellular association of HCV virion with lipoprotein components came from recent demonstrations that the density of viral particles is profoundly altered after incubation with lipid-rich media [59,60]. Indeed, the density of HCV-infected patient-derived viral particles evolve and become lower upon a lipid-rich diet of patients. This was confirmed in in vitro experiments by incubating high density HCVcc particles with human serum, which shifted them to lower densities (Figure 1B) and allowed their association with apoB, like for HCV derived from patients. It was also shown that VLDL but also HDL or LDL, which are not synthetized by the liver, are able to transfer lipids to viral particles, in a manner dependent on the presence of specific serum factor(s), which reinforced the idea that the association of particles with circulating neutral lipids is a dynamic and/or post-egress process. In line with this, other reports indicated that apolipoproteins, especially apoE, can be exchanged or transferred from lipoproteins, cell supernatant or serum to isolated infectious particles [61,62,63]. Finally, the density of purified viral particles could be also modified by lipoprotein lipase (LPL) or by hepatic triglyceride lipase (HTGL) [64], suggesting that the density of particles could result from a balance between association with lipids vs. their degradation, in a manner likely depending on the composition of the environment and specific factors.

In line with these observations, reestablishing a normal lipoprotein secretion profile in Huh-7.5 cells could restore the low-density profile of infectious particles, which, since sufficient lipid levels are secreted in these conditions, may occur in the extracellular medium as well as inside the producer cells [18]. Likewise, inhibition of cellular factors involved in the VLDL biogenesis pathway, like ACAT [48,49], could modulate maturation of particles, which could occur either inside the producer cells via a direct action during assembly/secretion or, alternatively, through modifications of the quantity and quality of secreted lipoproteins. 

### 4.3. Proposed Model for Extracellular Association of HCV Particles with Neutral Lipids

All these evidences indicate that independently of a virion lipidation process that can occur within the cells, which could depend on their capacity to secrete lipoproteins and/or required factors, the association of HCV particles with lipids is a dynamic process that evolves depending on the availability of lipids and specific proteins in the blood (Figure 3B). Indeed, HCV particles, already associated with neutral lipids or not, are secreted by hepatocytes. When they circulate in the extracellular milieu, they may encounter lipoproteins and serum proteins or factors, secreted by hepatocytes or by other cell types. Depending on the combination of these different components, particles could maturate and become more lipidated.

## 5. Regulators of HCV Association with Apolipoproteins and Lipids

### 5.1. The HyperVariable Region 1 of E2

The first 27 amino acids of the N-terminal part of E2 were identified as a sequence of high variability, which was termed hypervariable region 1 (HVR1). The functions of this domain have been thoroughly studied and, more particularly, it was shown that HVR1 regulates the lipidation of particles. First, HCV particles produced with a genome deleted from HVR1 display a higher density profile, between 1.10–1.16 [23,65,66]. In addition, point mutations in HVR1, i.e., either I399T (JFH1) [67] or L399R (H77) [23], were shown to alter the density of HCV particles. More recently the comparison between two strains of HCVcc, namely Jc1 and H77, which have different HVR1 sequences, confirmed that HVR1 acts as a regulator of the association of particles with lipids in a sequence specific manner [59]. Indeed, a chimeric Jc1 genome harboring HVR1 from H77 strain exhibited a lipidation profile resembling that of the H77 virus and a similar result was reciprocally found for a recombinant H77 virus harboring Jc1 HVR1, i.e., a profile close to that of Jc1.

Besides its involvement in virion association with lipids, HVR1 is also involved in the recruitment and conformation of some apolipoproteins incorporated at the surface of the HCV particles [61,68]. Indeed, while deletion of HVR1 did not influence the incorporation of apoE at the surface of particles, this influenced the neutralization profile achieved with anti-apoE antibodies.

### 5.2. Other Regions of E2

In addition to HVR1, a recent mutagenesis analysis of HCV E2 revealed that other regions of this glycoprotein could influence the density of the particles and hence, their lipidation. Particularly, one such mutant, i.e., G451R, was shown to increase the density of the viral particles [69,70] whereas another one, i.e., I414T, yielded HCV particles harboring a more heterogenous density that had higher infectivity in the low densities [71,72].

All in all, these studies indicated that E2, via its HVR1 determinant as well as other domains, is a major viral factor regulating the association of HCV particles with lipoprotein components.

### 5.3. Serum Factors

Incubation of non-lipidated HCV particles with individual lipoproteins, i.e., VLDL, LDL or HDL, revealed that viral particles are able to associate with either class of lipoprotein although the lipidation level is greater when the lipoproteins are combined [59]. In addition, this study revealed that virion lipidation by these lipoproteins also requires specific, non-lipid serum factors to help the process. Indeed, the environment provided by a lipoprotein-deficient serum (LPDS) was required to allow the association of HCV particles with purified lipoproteins. Interestingly, serum proteins that are involved in the exchange of neutral lipids between lipoproteins, such as cholesteryl ester transfer protein (CETP) and lecithin-cholesterol acyltransferase (LCAT), may not be involved in the association of HCV particles with lipids, as shown by using specific inhibitors of the aforementioned lipid transfer proteins. In contrast, human serum albumin (HSA) was shown to help the lipidation process by VLDL and LDL, although alternative factors seem to promote lipidation by HDL [59]. A proposed mechanism is that HSA could act by bridging apoB-containing lipoproteins and virions [73] or by favoring lipid efflux between the two partners [74].

As the concept of lipidation or maturation of particles occurring outside the cells is recent, more studies are required to fully understand the complete process of lipidation.

## 6. HCV Particles: One Particle or Two Particles Models?

Despite recent advances in the unraveling of the lipidation mechanism, the structure and properties of the association of HCV particles with neutral lipids and apolipoproteins remains poorly defined. It has been proposed that HCV LVPs may incorporate lipoprotein components through two different models. A first possibility is a direct exchange with lipoproteins or, alternatively, a fusion between viral particles and lipoproteins, allowing the formation of the so-called “one-particle model” [8,75], which could correspond to a viral particle embedded into a lipoprotein (Figure 1A). A second possibility is a non-covalent interaction of viral particles with lipoproteins, which involves interactions between E2, on the HCV particle side, and apoE, on the lipoprotein side, leading to the “two-particles model” [75] (Figure 1A). This second possibility could correspond to the association of viral particles with intact lipoproteins or, alternatively, with a “lipoprotein-like-vesicle” containing lipoprotein-derived neutral lipids and apolipoproteins with a composition regulated by the virus itself (see the two proposed models in Figure 1A). Interestingly, kinetic studies of incubation of particles with human serum indicated that lipidation is progressive [59], arguing against a direct association with a single, intact lipoprotein and reinforcing the idea of a dynamic process of association. Alternatively, it is possible that particles could associate progressively with several canonical lipoproteins (see the different proposed models in Figure 1A).

Recent electron microscopy (EM) pictures show that HCV particles are heterogenous in size [17,76] and have a non-regular structure, which reflects different compositions in term of proteins and lipids. These pictures may argue for the “one-particle” model [8,12,17,76] since the particles appear with a central electron dense disc and an external electron-light ring, the latter one being larger for HCV particles derived from patient’ sera [76]. However, since lipidation can evolve in the extracellular medium, as previously discussed, this does not exclude the “two-particles” model that may exhibit more transient or unstable properties. Alternatively, the latter particles detected by EM could have been less well captured by the antibodies used in these EM studies. Although it is also possible to imagine a combination of both models, the most important point to keep in mind is that HCV particles can evolve in the extracellular medium [59,60], which rules out that there is a unique structure for this virus.

## 7. Impact of Association of HCV Particles with Lipoproteins Components

### 7.1. Increase of Specific Infectivity

Particles in the low-density fractions are known to be the most infectious in vivo [9,10]. In line with this, in vitro models allowing the restoration of their lipidation showed an increased specific infectivity of low-density HCV particles [18,59]. However, the mechanism of this increased specific infectivity is not fully determined and may result from a different usage of cell surface receptors during entry. Indeed, the HCV entry process requires several lipoproteins receptors like the scavenger receptor B-I (SR-BI) [77], LDL-receptor (LDL-R) [78] or VLDL-receptor (VLDL-R) [79,80]. Interestingly, it was shown that depending on the density of the particles, the usage of SR-BI [23] as well the involvement of other lipoproteins receptors [79] are changed, which argues for a more efficient usage or requirement of the receptors during entry of lipidated particles. Alternatively, it was also shown that HDL [81,82,83,84,85,86] but also apoE [62,63] or apoC-I [61] enhance in trans HCV entry of HCV pseudo-particles (HCVpp) as well as of HCVcc, suggesting an action of HDL or of lipid-free exchangeable apolipoproteins in cell entry, independent of their possible association with viral particles. Therefore, the presence of HDL and/or apoE/apoC-I in virus inocula could also be responsible for the increased specific infectivity. More studies need to be performed to further understand these interesting observations.

### 7.2. Stability

HCVcc particles, produced in classical conditions that do not allow their lipidation, are stable at 4 °C [59]. Yet, previous reports indicated that the half-life of the virus decreases drastically in a temperature-dependent manner above room temperature [87]. Interestingly, the association of viral particles with lipids was shown to increase the virus half-life at 37 °C [18], suggesting that lipidation protects particles from degradation.

### 7.3. Protection against Neutralizing Antibodies

Early studies on patient-derived virus demonstrated that apoB-associated HCV particles failed to be precipitated with IgG [88], indicating that the association of particles with lipoproteins may contribute to the shielding of neutralizing epitopes. This notion of lipidation-induced shielding was confirmed in in vitro experiments that highlighted a correlation between the density of viral particles and their protection against neutralizing antibodies [70]. More recently, a direct comparison study using in vitro fully lipidated particles showed that the latter were far more resistant to several neutralizing antibodies as compared to non-lipidated particles [59]. Interestingly, HVR1 has been shown to be an important viral determinant mediating protection against neutralizing antibodies [65,66,83]. Since HVR1 also modulates the lipidation of particles (reviewed in [89]), it is tempting to speculate that HVR1 could influence virus protection by modulating the lipidation of particles. Besides neutral lipids, apoE is another factor that modulates the protection against neutralizing antibodies. Production of HCVcc in cells that do not express apoE resulted in particles with less incorporated apoE that were more sensitive to neutralizing antibodies [90]. The role of apoE in protection was further confirmed by others, since incubation of HCVcc particles with apoE resulted in a protection against different anti-E2 antibodies [63]. It is possible that apoE induces this protection by increasing the rate of cell entry [63] as well as by inhibiting Ficolin-2, an inhibitor of the attachment of particles to the cell surface [91].

Altogether, these results indicated that lipoproteins components associated with infectious particles are acting for immune evasion of the virus.

## 8. Conclusions

HCV particles are unique by their composition and structure since they resemble lipoproteins with a neutral lipid core and incorporation of apolipoproteins. Because of this particular shape, the rationale design of a vaccine remains very challenging. HCV morphogenesis is known to be highly linked to lipoprotein metabolism and recent results suggested that the composition of HCV particles in lipids and apolipoproteins were highly dynamic and depended on the environment. In addition, recent advances have highlighted methods to produce HCV particles in vitro with a composition that is identical to that of patient-derived HCV particles. This will be useful to better appreciate how infectious particles can evolve in lipid-rich environments as well as for understanding their maturation process and the humoral immune evasion mechanisms. Finally, as lipoprotein-association can shape the surface of viral particles and induce neutralization resistance, elucidating their structure would improve the design of rational vaccine candidates.
